# Taming the Unknown Unknowns in Complex Systems: Challenges and Opportunities for Modeling, Analysis and Control of Complex (Biological) Collectives

**DOI:** 10.3389/fphys.2019.01452

**Published:** 2019-12-03

**Authors:** Paul Bogdan

**Affiliations:** Ming Hsieh Department of Electrical and Computer Engineering, University of Southern California, Los Angeles, CA, United States

**Keywords:** fractals, time-varying complex networks, cyber-physical systems, network physiology, multifractal profile optimal control, unknown unknowns, causal predictive modeling, compact mathematical modeling

## Abstract

Despite significant effort on understanding complex biological systems, we lack a unified theory for modeling, inference, analysis, and efficient control of their dynamics in uncertain environments. These problems are made even more challenging when considering that only limited and noisy information is accessible for modeling, which can prove insufficient for explaining, and predicting the behavior of complex systems. For instance, missing information hampers the capabilities of analytical tools to uncover the true degrees of freedom and infer the model structure and parameters of complex biological systems. Toward this end, in this paper, we discuss several important mathematical challenges that could open new theoretical avenues in studying complex systems: (1) By understanding the universal laws characterizing the asymmetric statistics of magnitude increments and the complex space-time interdependency within one process and across many processes, we can develop a class of *compact yet accurate mathematical models* capable to potentially providing higher degree of predictability, and more efficient control strategies. (2) In order to better predict the onset of disease and their root cause, as well as potentially discover more efficient quality-of-life (QoL)-control strategies, we need to develop mathematical strategies that not only are capable to discover causal interactions and their corresponding mathematical expressions for space and time operators acting on biological processes, but also mathematical and algorithmic techniques to identify the number of unknown unknowns (UUs) and their interdependency with the observed variables. (3) Lastly, to improve the QoL of control strategies when facing intra- and inter-patient variability, the focus should not only be on specific values and ranges for biological processes, but also on optimizing/controlling knob variables that enforce a specific spatiotemporal multifractal behavior that corresponds to an initial healthy (patient specific) behavior. All in all, the modeling, analysis and control of complex biological collective systems requires a deeper understanding of the multifractal properties of high dimensional heterogeneous and noisy data streams and new algorithmic tools that exploit geometric, statistical physics, and information theoretic concepts to deal with these data challenges.

## Introduction

Genomic, proteomic, and physiological processes are generally used for medical diagnosis because they encompass the complex dynamics and multi-scale interactions between the chemical, electrical, and mechanical components of the human body. They exhibit higher-order statistical variability from person to person due to individual biological features (e.g., body mass and height) while also being highly influenced by a wide web of environmental factors (e.g., temperature, noise pollution, cultural traits, and social anxiety levels). Rigorous mathematical analysis shows that many such genomic, proteomic and physiological processes possess time dependent, long-range dependence, and multi-fractal characteristics (Goldberger and West, 1987; Ivanov et al., 2001; Wink et al., 2008; Bassingthwaighte et al., 2013; Ghorbani and Bogdan, 2013; Bohara et al., 2017; Akhrif et al., 2018; Ghorbani et al., 2018; Racz et al., 2018b). For instance, Lombardi et al. (2019) demonstrated that the existence of long-range temporal correlations (dependence) is an accurate marker of “healthy brains.” Moreover, mathematical investigations of physiological processes collected from the individuals suffering from various diseases revealed specific patterns, for example, a decrease in correlation in both temporal and fractal behavior (Ivanov et al., 1999; Stanley et al., 1999; Kotani et al., 2005; Gierałtowski et al., 2012). For instance, the ratio between the short-term and long-term scaling exponents was demonstrated in Platiša et al. (2019) to discriminate between patients experiencing heart failure, providing crucial information where the levels of the cardiac autonomic nervous system control, age, or the left ventricular ejection fraction could not. Similarly, fractal scaling has been demonstrated not only to be capable to discriminate between type 1, type 2 diabetes, and non-diabetic subjects, but also identify the dynamical instabilities in the glucoregulation (Kohnert et al., 2018).

Despite this significant body of work, current diagnosis methods and medical devices (e.g., pacemakers, artificial pancreas, anesthesia systems, and brain-machine-body interfaces) do not account for these mathematical characteristics, thus perpetuating a superficial understanding and deciphering of the unknown unknowns (UUs) governing their complex dynamics and possibly leading to a lower quality-of-life (QoL). Consequently, through this position statement, we aim to catalyze a shift of paradigm by calling for a mode of personalized and precise medicine that is more patient (and physiological complexity aware) centered and which does not rely on generic signal reference values that are patient independent. This new rigorous mathematical and algorithmic paradigm needs to be integrated into future smart medical cyber-physical systems (MCPS) in order to facilitate effective patient-centered healthcare to improve current delivery of care and cut down on its high costs. The MCPS design (Lee et al., 2012) – integrating sensors for assessing (computing/mining) individual physiological state, communicating this information via a network infrastructure from home-to-hospital to medical experts, and controlling vital health signals (e.g., cardiac pacing, insulin level, blood pressure, and brain activity) to prevent health complications, maintain good health, and/or avoid fatal conditions – require a cross-disciplinary approach.

Toward achieving the design of these genomic, proteomic, and physiological complexity-aware MCPS architectures, in this paper, we briefly review a list of urgent mathematical challenges and advocate for (1) a comprehensive understanding of individual complexity of genomic, proteomic and physiological processes in order (2) to establish compact yet accurate mathematical models (Xue and Bogdan, 2017) to predict abnormal behavior corresponding to disease precursor patterns, and (3) to optimize the dynamics of human physiology in accordance with observed complexity (minimize detrimental effects on homeostasis that potentially minimizes also the healthcare costs) and improve the patients QoL. These envisioned complexity-aware MCPS are bound to exploit the fractal geometry, non-linear dynamics, fractional calculus, fractal statistics, and stochastic fractal optimal control for maximizing the impact of prevention, treatment, and QoL, while minimizing health care costs related to hospitalization or side-effects. Also, these MCPS should prevent misuse, overuse or underuse of medical care based on robust mathematical analysis, thus cutting down on healthcare costs, and improving efficiency.

## Biological (Genomic, Proteomic, Physiological) Complexity: Modeling, Analysis, and Control

### Genomic, Proteomic, and Physiological Signals Display Asymmetric Non-Gaussian Dynamics

**FIGURE 1:**
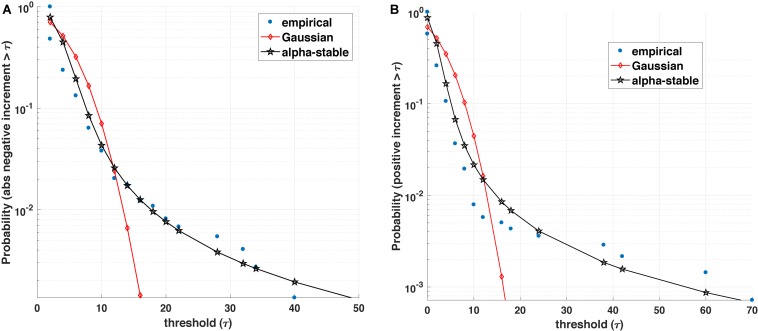
Blood glucose analysis. **(A)** Empirical value of the survival function (i.e., the probability that the positive increment exceeds a threshold τ) is better represented by an α-stable distribution than a Gaussian counterpart. **(B)** Similarly, the empirical survival function for absolute value of negative increments with varying threshold τ is better represented by an α-stable than a Gaussian distribution.

While many pioneering and recent studies have demonstrated that genomic, proteomic and physiological processes possess self-similar, long-range dependence (memory), and fractal characteristics (Goldberger and West, 1987; Shlesinger, 1987; Ivanov et al., 1999, 2001; Stanley et al., 1999; Eke et al., 2006; Bassingthwaighte et al., 2013; Ghorbani et al., 2018; Racz et al., 2018b), an intriguing mathematical observation is that many such physiological processes display an asymmetric non-Gaussian dynamics. From a mathematical perspective, this implies that the dynamics of the process under investigation is governed by two components (see Figure 1): the magnitude of positive and negative increments and the inter-event (waiting times between events) of a biological process.

The statistics of the magnitude of positive and negative increments of a process can encode information about the degree of asymmetry, non-linearity, and the influence of other variables on its dynamics. Many complex (biological) processes exhibit positive (growth) and negative (decline/loss) jumps or bursts characterized by different distributions (e.g., stretched exponential and asymmetric α-stable distributions). For instance, Figure 1 illustrates that most often the positive and (absolute values of) negative increments in blood glucose are better fitted by an α-stable distribution family than exponential or Gaussian distributions. Within this context, a number of open questions arise: (1) From a medical perspective, can the observed asymmetric α-stable statistical behavior represent a universal behavior of a healthy dynamics for specific biological processes? If yes, can the potential deviations in the asymmetric α-stable statistical behavior (e.g., decrease in the degree of asymmetry, decrease/increase in the α-stable parameter) be linked with the disease precursors? Along the same lines, how does the observed asymmetric α-stable statistical behavior evolve with aging? For example, as pointed out in Ferrucci et al. (2018), aging contributes to the stiffening of heart ventricles and large arteries, which leads not only to detrimental changes in cardiovascular performance and physical capacity, but could also influence the statistics of many physiological processes (e.g., heart rate, blood glucose, and brain activity processes). (2) From a mathematical perspective, one can wonder whether the statistics of the magnitude increments can shed light not only on the mathematical expressions (e.g., linear, quadratic, and fractional order) describing the rate of change of one state variable, but also on the causal influence and its corresponding mathematical terms characterizing the coupling among the state variables of the physiological systems. In other words, rather than postulating possibly unjustifiable mathematical expressions and using these postulates for formulating inverse problems, a more rigorous mathematical analysis of physiological complexity would require to carefully analyze the statistics of the positive and negative magnitude increments and encapsulate the statistical findings into generative mathematical models [e.g., generalized master equations (Kenkre et al., 1973; Klafter et al., 1987; Balescu, 1997; Akhrif et al., 2018)].

Moreover, the statistics of time-intervals at which a process changes its value (inter-event or waiting times) dictates whether the process under investigation is possessing short-range dependence (Markovian) or long-range dependence (non-Markovian) characteristics. For exponential inter-event times, the rate of change in the variable can be described by a first order time derivative. In contrast, for single power law distributed inter-event times, the rate of change in one state variable requires the introduction of a fractional order derivative (Shlesinger, 1987; West, 2010; Svenkeson et al., 2016). Of note, as demonstrated in Lombardi et al. (2019), the distribution of inter-event times (among successive events) can not only shed light on the nature of the operator governing the rate of change (dynamics), but also allow us to study the hierarchical temporal organization of the neuronal avalanches (i.e., an ensemble of neurons that fire close-in-time) and the existence of a critical behavior. Nevertheless, the set of critical exponents characterizing the neuronal spontaneous activity in control conditions and in the presence of folic acid are different (Yaghoubi et al., 2018) suggesting the existence of different universality classes. Consequently, a more challenging problem is whether for a process with multi-modal power law distributed inter-event times, the rate of change in the state variable can be accurately described by a combination of fractional order derivatives [i.e., *d^α^ x*(*t*)/*dt*^α^ = *D*^α^ *x*(*t*), α being the fractional exponent of the fractional derivative] in order to capture the complex memory structure, and how this is connected with criticality (Xue and Bogdan, 2017). Concomitantly, there is a need for mathematical and medical research to understand the statistical complexity of such inter-event times: From a mathematical and bio-physics perspective, we need to better understand the phase transition phenomena characterized by the emergence of multi-modal power law distributed inter-event times and correlate there observations with the degree of the robustness, self-organization, biological intelligence/adaptivity, stability, resiliency, and efficiency of a dynamical system. From a medical perspective, we need to elucidate the relationship between a single or a multi-modal power law distributed inter-event times and the healthy critical or pathological brain states. Future research needs to investigate the relationship between the statistical properties of the magnitude increment and inter-event times and correlate it with the specific mathematical structure of the dynamical equations. Moreover, a more comprehensive cyber-physical systems research is required to analyze genomic, proteomic, and physiological processes in order to elucidate the effect of aging on the relationship between the statistical properties of the magnitude increments and inter-event (waiting) times and determine if changes in these statistical properties can be associated with precursors of diseases. Understanding the universal statistical properties of healthy biological systems as well as healthy aging, could contribute to identifying the disease markers (e.g., detecting genomic instability, epigenetic alterations, mitochondrial dysfunction, and loss of proteostasis), and defining new molecular-based or cellular-based control strategies to correct unhealthy courses and potentially delay or avoid the onset of diseases (Ferrucci et al., 2018).

### Biological Systems Display a Complex Spatio-Temporal Interdependent Dynamics Subject to Unknown Unknowns

**FIGURE 2:**
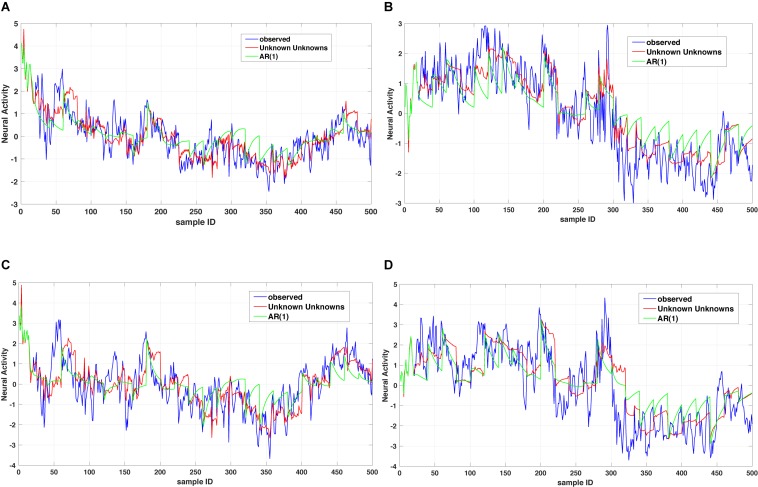
Unknown unknowns (UUs). Twenty step prediction comparison of two models, a multi-dimensional fractional dynamic model with UUs and a multi-dimensional memoryless dynamic model (termed one leg autoregressive AR(1). **(A,B)** shows comparison of one channel across different time windows, with AR(1) always overshooting the prediction. Similarly, **(C,D)** are representing the comparison of another channel with two time windows and as it can be noticed the AR(1) exhibits several overshoot/undershoot events in the prediction as compared to the multidimensional fractional dynamic model with UUs.

The ability to efficiently (in real-time) analyze and extract information from large-scale biological datasets is essential for inferring the complex interdependency and corresponding multi-variable functional dependency among various genomic, proteomic and physiological processes, determining the types, and number of required operators (i.e., spatiotemporal integer or fractional order integrals or derivatives) to describe the observed dynamics, incorporating realistic features into compact dynamic models and for endowing MCPS with cognition and intelligence. Current mathematical approaches (e.g., machine learning and system identification) build such dynamic models on simplistic or unverified assumptions (e.g., Markovian dynamics) and achieve good accuracy/fidelity by increasing the number of parameters and the modeling complexity. Such methodologies may pose not only computational challenges, but also impede our understanding of complex biological systems and the design of accurate MCPSs (e.g., brain-machine interfaces, bionic systems) (Pequito et al., 2015; Gupta et al., 2019). One simple approach to account for the spatial complex time-varying interdependency between biological processes and for their short- or long-range memory properties is to construct mathematical models of the following form (Xue et al., 2016b):

(1) $$\left[\begin{array}{c} \frac{d^{\alpha_{1}}\,x_{1}\,\left(t\right)}{d\,t^{\alpha_{1}}}\\ \vdots \\ \frac{d^{\alpha_{n}}\,x_{n}\,\left(t\right)}{d\,t^{\alpha_{n}}} \end{array}\right]=\sum_{p=1}^{M}A_{p}\,\left[\begin{array}{c} x_{1}\,\left(t-\tau_{p}\right)\\ \vdots \\ x_{n}\,\left(t-\tau_{p}\right) \end{array}\right]+E\,\left(t\right)$$

where *x*_1_(*t*),…,*x*_*n*_(*t*) denote a set of biological processes, *A*_*p*_ represents a coupling matrix encoding the linear interdependencies at previous time points *t*−τ_*p*_ and *E*(*t*) denotes an *n*-dimensional error term (Xue et al., 2016b). In Eq. (1), the dynamics of a biological process *x*_*k*_(*t*) is governed by a general operator *d^α^ x*(*t*)/*dt*^α^ = *D*^α^ *x*(*t*) and based on the observed dynamics *x*_*k*_(*t*) can be coupled to all other dependent processes *x*_1_(*t*),…,*x*_*n*_(*t*) or a subset of those. The time operator can either reduce to an integer order derivative for capturing short-range memory dynamics or a fractional order derivative for capturing long-range memory dynamics. For instance, Figure 2 shows a comparison between two dynamic models of the type summarized in Eq. (1) for the case of considering a memoryless (integer order) example and a fractional order one. As one can notice the multi-dimensional fractional order dynamical model of the type summarized in Eq. (1) provides a better prediction when compared to the multidimensional integer order counterpart.

Consequently, one computational benefit of dynamic models represented in Eq. (1) is that by exploiting up to *n* fractional order coefficients and one coupling matrix, we can achieve a more compact mathematical representation of complex biological systems with better prediction accuracy than considering higher order autoregressive integrated moving average models. An alternative data-driven mining of the complexity of stochastic processes can be achieved by investigating and estimating the entropy, conditional entropy and information storage of their realizations (Xiong et al., 2017). Although the analysis in Xiong et al. (2017) highlights the importance of non-stationarities (i.e., trends, spikes, and local variance change) and the long-range correlations on the complexity of stochastic autoregressive processes, it also demonstrates that appropriate preprocessing is critical for employing the entropy-based algorithms for mining physiological states, discriminating among various degree of disease, and identifying urgent clinical conditions. Consequently, there is an urgent need for developing algorithmic tools that combine the compact yet accurate mathematical modeling (as the one above-mentioned) with entropic measures for quantifying the complexity of multi-dimensional dynamical systems. From a multiscale multi-dimensional perspective, the multiscale Granger causality was introduced in Faes et al. (2017) to quantify the information transfer across multiple time scales and assess the directed lagged interactions among joint processes (represented by time series). These pioneering works make fundamental contributions toward understanding the multiscale causal relationships among coupled stochastic processes that could be further investigated and extended within the context of Eq. (1) or develop data-driven learning strategies for identifying the structure of Eq. (2).

Along these lines, a major mathematical challenge is how to optimally sense spatio-temporal interdependent cyber-physiological processes exhibiting both short- and long-range memory properties (mainly concerning the dynamics) or short- and long-range cross-dependence properties (concerning the interdependence structure among processes). Alternatively stated, the critical sensing deployment problems seek to determine the minimum number of sensors and their spatial deployment in order to minimize the state estimation error and the process disturbance (Pequito et al., 2015; Xue et al., 2016a; Tzoumas et al., 2018). These problems are even more challenging when considering the intra- and inter-patient variability. For instance, a compact dynamical model as the one in Eq. (1) can be exploited not only for studying such observability related problems, but can also provide new insights into the poorly understood neuro-activation dynamics of motor-related tasks or can suggest design strategies for MCPS (such as the EEG-wearable systems). It is required that such algorithmic strategies are capable of not only capturing the intrinsic spatiotemporal fractality of MCPS through compact mathematical models (with fewest number of parameters), but also allow us to retrieve and predict the states of complex biological systems from small collection of measurements. Although determining the smallest number of sensors required to ensure the observability and retrieve the overall evolution of a coupled fractional and integer order dynamical systems [e.g., collections of electroencephalogram (EEG), electromyogram (EMG), or electrocardiogram (ECG) signals] relied on submodular optimization (Pequito et al., 2015; Xue et al., 2016a; Tzoumas et al., 2018), further studies should investigate non-submodular optimization strategies.

While dynamic models of the type in Eq. (1) can provide compact mathematical representation of complex brain activity and brain-muscle interdependent networks, a much more general mathematical representation may take the following form:

(2) $$\int_{\alpha_{m\,i\,n}}^{\alpha_{m\,a\,x}}h\,\left(\alpha_{k}\right)\,\frac{d^{\alpha_{k}}\,x_{k}\,\left(t\right)}{d\,t^{\alpha_{k}}}\,d\alpha_{k}=F\,\left(x_{1},\ldots \,x_{k},\ldots \,x_{n};t\right)+E\,\left(t\right)$$

where *h*(α_*k*_) represents a distribution of fractional order exponents for a specific range α_*min*_≤α_*k*_≤α_*max*_. Such a distribution *h*(α_*k*_) may be introduced to model biological processes that display a multi-fractal behavior requiring multiple fractional order derivatives for modeling their dynamic behavior. The function *F*(*x*_1_,…*x*_*k*_,…*x*_*n*_; *t*) in Eq. (2) encodes the interactions among various processes and can be used to obtain a complex network representation of the physiological systems. For instance, pioneering efforts (Ivanov and Bartsch, 2014; Bartsch et al., 2015; Liu et al., 2015; Ivanov et al., 2016) have demonstrated that various physiological processes can be described through a complex network approach (i.e., the network physiology; Bashan et al., 2012). By exploiting the time delay stability concept, the authors in Ivanov and Bartsch (2014) and Ivanov et al. (2016) quantified the dynamic links among physiological systems and demonstrated a robust relation between the network structure and the physiological states. Moreover, despite numerous studies demonstrating the multi-fractal behavior of various biological processes (Goldberger and West, 1987; Stam and de Bruin, 2004; Wink et al., 2008; Bassingthwaighte et al., 2013; Delignières et al., 2016; França et al., 2018; Mukli et al., 2018; Racz et al., 2018a; Wendt et al., 2018), we lack mathematical and algorithmic tools for identifying the causal interdependence structure and the parameters of dynamical models of the type in Eq. (2). Identifying the mathematical expressions of the functions *F*(*x*_1_,…*x*_*k*_,…*x*_*n*_; *t*) and reconstructing the physiological networks may be challenging not only because we are required to process heterogeneous, multimodal, and noisy time series (representing various complex multi-component dynamical systems with their own regulatory mechanisms) corresponding to different types of nodes (Ivanov and Bartsch, 2014; Ivanov et al., 2016), but also due to missing samples or scarce observations. Traditional regression techniques may not prove useful and new inverse problems [that may consider extensions of time delay stability concept (Ivanov and Bartsch, 2014; Ivanov et al., 2016)] need to be formulated and solved for identifying the true compact yet accurate mathematical models of biological systems. However, developing rigorous mathematical techniques for identifying the universal behavior encoded in the functions *F*(*x*_1_,…*x*_*k*_,…*x*_*n*_; *t*) from multimodal heterogeneous time series can not only help decipher the functionality associated with specific physiological network structures, but also develop strategies to detect the spatiotemporal emergence of phase transitions in physiological networks and identify early precursors of diseases and frailty.

Multi-scale multi-physics interactions lead to complex spatiotemporal interdependency and pose significant challenges for MCPS observability and their compact mathematical modeling. In many practical settings, the sensing of time-varying complex networks can only observe a small subset of nodes. Consequently, a major research challenge is on developing mathematical and algorithmic strategies that can tackle the following problems: (*i*) How to infer the number of unknown unknowns and the interdependency structure not only between the observed variables, but also between the observed and the inferred (unobserved) ones? (ii) How to identify the minimal subset of variables that need to be measured in order to retrieve the unknown CPS states and unknown inputs triggering the overall evolution? For instance, the necessary and sufficient conditions for ensuring the retrieval of state and unknown stimuli and an efficient algorithm to determine a small subset of variables that need to be measured for recovering the states and inputs while establishing sub-optimality guarantees with respect to the smallest possible subset were discussed in Gupta et al. (2018a, b). Exploiting these theoretical tools for identifying compact mathematical modeling while dealing with UUs, a rethinking of the design of EEG-based non-invasive brain machine interfaces (BMIs) was described in order to endow these BMI systems with new algorithmic strategies that identify the parameters of a fractal time-varying complex network for describing the interactions between various brain regions (Gupta et al., 2018a, b, 2019). The parameters of the compact mathematical model are used to decode the spatio-temporal fingerprints of human decision-making processes and classify specific cognitive states (e.g., motor task or its imagination) based on measurements collected from a brain in action and in context. The classification performance on real brain activity motor tasks datasets is on average 85.7% (Gupta et al., 2018b). Thus, this compact mathematical modeling provides excellent features for differentiating among various brain imagined motor movements.

Although promising, in general, the model structure dictated by the biological systems and the environmental influences are unknown. Future research needs to either develop strategies to account for the situation in which each physiological process is characterized by a distribution of fractional order coefficients or determine tradeoff laws that characterize the minimum number of fractional order coefficients that are required for accurate observability and prediction of the overall complex system dynamics. From a medical perspective, we need to investigate whether the above-mentioned compact mathematical modeling enables the definition of robust strategies to detect and identify the hallmarks of aging, or how aging phenotypes, age-related diseases and functional limitations (Ferrucci et al., 2018) influence the structure, fractal profiles and parameter ranges of this compact mathematical model. Similarly, a crucial step toward developing multiscale (long-term) control methodologies with minimal effort or intervention requires compact mathematical models extracted from scarce, sparse, heterogeneous and noisy data, and yet capable of predicting the likelihood of frailty and disability.

### Controlling Physiological Complexity

The goal of a MCPS is not only to monitor and construct a dynamical model of complex biological systems, but also to find adequate control strategies that maintain the physiological state within predefined healthy range while minimizing the adversarial effect of the control signal and so improve the QoL of patients (Bogdan et al., 2013; Ghorbani and Bogdan, 2013). For instance, the control algorithm of an artificial pancreas utilizes the model describing the blood glucose to insulin dynamics to determine the minimum amount of insulin to be injected at the prescribed times over a finite horizon of time such that the risk of hypo- or hyperglycemia is minimized (Ghorbani and Bogdan, 2013, 2014). Of note, it is important to find the insulin amount with respect to QoL constraints, because injecting too much too soon or too frequently can affect other organs and can have a detrimental health effect over longer periods of times (months and years). Given the observed physiological variability and taking into account recent findings that associate the loss in the degree of multi-fractal properties with a signature of frailty and departure from homeostasis toward a disease state, one naturally asks how the physiological control problems should be formulated within the healthcare CPS architectures. Alternatively stated, given the intra- and inter-patient variability, enforcing a specific physiological reference value without considering the multi-fractal characteristics may sometimes do more harm than good. It is becoming well accepted that the physiological control should obey a stealthy intervention or influence on the time-varying complex physiological networks (i.e., sparsest in time and minimum amount of actuation signal) such that the control effort does not destabilize the healthy functional feedback (regulatory) loops or contribute to a form of adaptation of the complex biological systems to the therapeutic agent. From this perspective, the physiological control in MCPS should not only consider healthy physiological ranges for state and control variables, but should also ensure that the healthy degree of multi-fractality of an individual is restored. While this mathematical problem remains to be carefully studied, it can be realized that one natural way to control the degree of multi-fractality and implicitly the physiological complexity measure is to optimize over the space of higher statistical moments and cross-moments associated with physiological networks.

To take into account the above-mentioned challenges, we hypothesize a physiological-aware control of complexity problem as a finite horizon stochastic optimization problem of the following form:

(3) $$m\,i\,n_{u\,\left(t\right)}\,\int_{t_{i\,n\,i\,t\,i\,a\,l}}^{t_{f\,i\,n\,a\,l}}\int_{x_{m\,i\,n}}^{x_{m\,a\,x}}C\,\left(t,\left\langle \left|x\right|\right\rangle ,{\left\langle \left|x\right|\right\rangle }^{2},\ldots ,{\left\langle \left|x\right|\right\rangle }^{k},u,r\right)\,dt\,dx$$

$$\frac{\partial^{\beta \,\left(t\right)}{\left\langle |x|\right\rangle }^{k}}{\partial t^{\beta \,\left(t\right)}}=\int_{\alpha_{m\,i\,n}}^{\alpha_{m\,a\,x}}\left\{A\,\left(k,\alpha \right)\,\left\langle {\left|x\right|}^{k-\alpha }\right\rangle +B\,\left(k,\alpha \right)\,\left\langle {\left|x\right|}^{k-2\,\alpha }\right\rangle \right\}\,g\,\left(\alpha \right)\,d\alpha$$

(4) $$+m\,\left(u,t\right)+h\,\left(\left\langle \left|x\right|\right\rangle ,\left\langle {\left|x\right|}^{2}\right\rangle ,\ldots ,\left\langle {\left|x\right|}^{k}\right\rangle ,t\right)\,\eta \,\left(t\right)$$

$$u_{m\,i\,n}\,\left(t\right)\leq u\,\left(t\right)\leq u_{m\,a\,x}\,\left(t\right)$$

(5) $$\left\langle {\left|x\right|}^{k}\right\rangle \,\left(t_{i\,n\,i\,t\,i\,a\,l}\right)=m_{i\,n\,i\,t\,i\,a\,l}^{k}\;k=1,2,\ldots \,N$$

where *C*(*t*, ⟨|*x*|⟩, ⟨|*x*|^2^⟩,…, ⟨|*x*|*^k^*⟩, *u*, *r*) denotes the cost objective as a function of the higher (first *k*-th) order moments [whose dynamics can be described by stochastic differential equations of the type in Eq. (4)], the control signals *u*(*t*) and the healthy physiological reference values *r*(*t*), *u*_*min*_ and *u*_*max*_ are the lower and upper bounds on the acceptable control signals *u*(*t*) in Eq. (5), β(*t*) is the time dependent fractal profile exhibited by the physiological process *x*(*t*), α and *g*(α) are the fractal exponent and distribution of fractal exponents characterizing the changes in the magnitude of stochastic (physiological) process *x*(*t*), *g*(*u*,*t*) denotes a function capturing the dependency between the *k*-th order moments and the control signals *u*(*t*), and *h* is a function meant to capture the additive or multiplicative nature of the noise sources η(*t*). The reason for accounting for various noise types is motivated by either measurement errors due to variations in the body posture and sensor transient malfunctioning, or communication failures and delays that can occur between various MCPS components. In Eq. (5), we denote by $\left\langle {\left|x\right|}^{k}\right\rangle \,\left(t_{i\,n\,i\,t\,i\,a\,l}\right)=m_{i\,n\,i\,t\,i\,a\,l}^{k}$ the initial values of the *k*-th order moments. Depending on the medical condition, the clinicians would not only enforce a specific mean for the physiological process, but also minimize the chances of rare events by considering the fourth-order moment or other related metrics. To account for the inter-patient variability, this framework allows us to characterize the statistical properties of a healthy person, derive a stochastic profile in terms of the *k*-th order moments, and use these models for maintaining an adequate physiological state.

The mathematical expression of the cost *C* in Eq. (3) depends on the physiological processes to be controlled, the coupled (interdependent) dynamics between the physiological and control signals and the medical condition to be treated. For instance, the cost function of the controller of an AP will have very different expressions when considering diabetes type or the lifestyle conditions such as blood glucose regulation during either nighttime or intensive exercise. From this perspective, there is an urgent need for developing mathematical and algorithmic control strategies for dealing with observed non-linear, time-varying, and inter- and intra-patient variability and encapsulate them into real-time physiological controllers. For example, it is important to determine how to best select a subset of control variables (e.g., single vs. dual hormone controllers for artificial pancreas) for regulating the physiological network in order to achieve the QoL control with minimal intervention (e.g., smooth BG). Equally important is the development of artificial intelligence and machine learning techniques for identifying the optimal risk indices to be optimized in order to provide quality-of-care control (prevent insulin overdose). Given the observed intra- and inter-patient physiological variability, the intelligence of MCPS should also be able to account for time-varying parameter uncertainty and modeled dynamics (unknown sensitivities to control variables), measurement and actuation delays, as well as the distributed nature of MCPS (distributed sensors and controllers). Having knowledge of the healthy physiological complexity of an individual (described through multi-fractal (Delignières et al., 2016), emergence (Balaban et al., 2018), self-organization (Balaban et al., 2018), and robustness metrics), can the MCPS controllers accurately determine (estimate) or retrieve the physiological state when facing sensor noise, adversarial events or actuator errors? Alternatively, can the MCPS controllers distinguish between sensor/actuator faults, abnormal medical conditions and external disturbances (e.g., mean, exercise, and stress levels)? While fractal research has contributed to solving anomaly detection problems in other application domains, we also need to consider how MCPS algorithms can exploit the genomic, proteomic and physiological multi-fractal and complexity to detect changes in the physiological state early on by correlating the mathematical characteristics with particular disease patterns. For instance, there is an urgent need for a comprehensive analysis of functional and phenotypic aging, as well as the development of algorithms for identifying the accelerated aging (Ferrucci et al., 2018), which can enable new control methodologies for delaying or avoiding frailty states. Preliminary research on the fractal physiology promises the design of future MCPS architectures that can determine the type of activity in which the patient is involved, monitor major physiological processes, locally regulate the multiscale physiological dynamics, and remotely inform clinicians via smart alerts.

## Conclusion and Summary

While complex networks have been recognized to model biological complexity and decipher medical therapeutics (Barabási et al., 2011; West, 2014; Udrescu et al., 2016), we still lack robust and rigorous data science and analytics techniques for mining the incomplete, heterogeneous and noisy biological data streams and extract their spatiotemporal interdependency. Relying on simplifying assumptions such as memoryless dynamics for either modeling biological processes or linearity for inferring the directionality of causal interactions can provide inaccurate inference strategies of the time-varying complex networks that govern the healthy dynamics of anatomical (biological) systems, which in turn can derail medical therapies. In contrast, by carefully investigating the fractal time properties of neural dynamics one can gain a better understanding and more accurate decoding of the human intent from EEG brain activity (Gupta et al., 2018b, 2019). At the same time, by carefully understanding the potential universal asymmetric statistical characteristics and their implications on the types of fractal (feedback) control architectures, that govern the healthy dynamics of biological processes, can not only provide a more accurate definition of homeostasis, but also open the avenue for new medical control strategies. However, these mathematical problems are made even more difficult when considering that some measurements may be incomplete (e.g., consist of missing contiguous sequences of measurements, measurements affected by noise, many important variables cannot be measured or are not known in order to be measured) or that we have access only to a few partially observable snapshots of the biological network that may suffer from environmental (malicious) interventions (e.g., manifestation of psychological stress and multiscale viral influences) (Xue and Bogdan, 2019; Gupta et al., 2019). Consequently, there is an urgent need for developing rigorous data science approaches that can accurately and efficiently mine the complex spatiotemporal interdependency among biological processes not only for constructing compact accurate mathematical models that can detect and predict abnormality but also for enabling more efficient control strategies to delay (or even avoid) frailty.

## Data Availability Statement

Publicly available datasets were analyzed in this study. This data can be found here: https://www.physionet.org/.

## Author Contributions

The author confirms being the sole contributor of this work and has approved it for publication.

## Conflict of Interest

The author declares that the research was conducted in the absence of any commercial or financial relationships that could be construed as a potential conflict of interest.
